# Transactions of State Association

**Published:** 1876-06

**Authors:** 


We learn from the Secretary of the State Association that he has not yet felt justified in commencing publication of the Transactions, though he has had the material ready for the printer for several weeks. He very rightly hesitates to contract the debt before there is reasonable assurance of funds forthcoming to meet it. It requires a considerable sum of money to print a volume of two or three hundred pages, together with heavy postage, and incidentals. We are assured though, that the work will be forwarded as rapidly as possible.

